# Ischemic Heart Disease Incidence in Relation to Fine versus Total Particulate Matter Exposure in a U.S. Aluminum Industry Cohort

**DOI:** 10.1371/journal.pone.0156613

**Published:** 2016-06-01

**Authors:** Andreas M. Neophytou, Elizabeth M. Noth, Sa Liu, Sadie Costello, S. Katharine Hammond, Mark R. Cullen, Ellen A. Eisen

**Affiliations:** 1 Division of Environmental Health Sciences, School of Public Health, University of California, Berkeley, California, United States of America; 2 Division of General Medical Disciplines, School of Medicine, Stanford University, Stanford, California, United States of America; Utah State University, UNITED STATES

## Abstract

Ischemic heart disease (IHD) has been linked to exposures to airborne particles with an aerodynamic diameter <2.5 *μ*m (PM_2.5_) in the ambient environment and in occupational settings. Routine industrial exposure monitoring, however, has traditionally focused on total particulate matter (TPM). To assess potential benefits of PM_2.5_ monitoring, we compared the exposure-response relationships between both PM_2.5_ and TPM and incidence of IHD in a cohort of active aluminum industry workers. To account for the presence of time varying confounding by health status we applied marginal structural Cox models in a cohort followed with medical claims data for IHD incidence from 1998 to 2012. Analyses were stratified by work process into smelters (n = 6,579) and fabrication (n = 7,432). Binary exposure was defined by the 10^*th*^-percentile cut-off from the respective TPM and PM_2.5_ exposure distributions for each work process. Hazard Ratios (HR) comparing always exposed above the exposure cut-off to always exposed below the cut-off were higher for PM_2.5_, with HRs of 1.70 (95% confidence interval (CI): 1.11–2.60) and 1.48 (95% CI: 1.02–2.13) in smelters and fabrication, respectively. For TPM, the HRs were 1.25 (95% CI: 0.89–1.77) and 1.25 (95% CI: 0.88–1.77) for smelters and fabrication respectively. Although TPM and PM_2.5_ were highly correlated in this work environment, results indicate that, consistent with biologic plausibility, PM_2.5_ is a stronger predictor of IHD risk than TPM. Cardiovascular risk management in the aluminum industry, and other similar work environments, could be better guided by exposure surveillance programs monitoring PM_2.5_.

## Introduction

Exposure to airborne particulate matter has been linked with increased risk of cardiovascular disease in the general population and more recently in occupational settings [1–6]. Particles of different sizes have different magnitudes of effect on cardiovascular health, with particulate matter with an aerodynamic diameter of less than 2.5 *μ*m (PM_2.5_) generally more strongly associated with adverse cardiovascular outcomes than larger particles [1, 7, 8].

The U.S. Environmental Protection Agency (EPA) began regulating air quality standards for Total Suspended Particles (TSP) in 1971, and with the advent of new monitoring technologies and increasing evidence for the health effects of smaller particles, more likely to reach the lower regions of the respiratory tract, later begun regulating smaller particles. It introduced limits focusing on particles defined as particulate matter with an aerodynamic diameter of less than 10 *μ*m (PM_10_) in 1987 and finally first adopted standards for PM_2.5_ in 1997 [9] (a fraction focusing mostly on combustion particles).

While most of the evidence of the adverse cardiovascular health effects of particulate exposures comes from general population studies of ambient air pollution, there have also been links reported in industrial settings, including the aluminum industry [4–6, 10, 11]. Exposures in occupational settings occur at much higher concentrations than ambient levels. Occupational exposure standards are usually based on composition and toxicity of specific exposure constituents rather than mass concentration or particle size. The Occupational Safety and Health Administration (OSHA) has standards for “particulates not otherwise regulated” with a Permissible Exposure Limit (PEL) of 15 mg/m^3^ for total dust, and 5 mg/m^3^ for the respirable fraction (defined as the sub fraction of inhaled particles that penetrates into the alveolar region of the lung [12]). Cal OSHA requires and ACGIH recommends, a lower limit of 10 mg/m^3^ for total and inhalable dust (defined as the fraction of airborne particles that enters the body through the nose and mouth corresponding to particles with aerodynamic diameter no greater than 100 *μ*m [12]) respectively, referencing eye, skin and respiratory irritation as relevant health effects [13]. Those values are orders of magnitude higher than comparable ambient air pollution standards.

We have previously reported increased risk of ischemic heart disease (IHD) related to PM_2.5_ exposures in a cohort of hourly workers in aluminum industry facilities [14–16]. Routine monitoring data for total particulate matter (TPM) were supplemented by research based PM_2.5_ measurements and exposures to both were used to create a job exposure matrix (JEM) [17]. We examined the relationship between TPM and PM_2.5_ concentrations in the JEM and contrast the two exposures as predictors of risk for incident ischemic heart disease (IHD) in this cohort.

## Methods

### Study Population

The study was approved by the institutional review boards of the participating institutions (University of California Berkeley and Stanford University). No informed consent was obtained as all data were de-identified and analyzed anonymously. The cohort has been described in detail elsewhere [14]. Briefly, health data were collected from hourly workers at 11 US facilities of one aluminum company, during the period from January 1, 1996 through December 31, 2012. Eligibility criteria included enrollment in the company x2019;s primary insurance plan and at least two years of employment as an hourly worker during the follow-up period. A two-year washout period was also implemented to rule out any prevalent ischemic heart disease cases. Follow-up began in 1998 (after the two-year washout period implementation), or January 1, 2003 for two of the participating facilities, due to a later acquisition of the facilities by the company. Subjects contributed person time beginning at start of follow-up or two years after their hire date if that came later than the start of follow-up. Participants were classified as either smelter or fabrication workers based on their work process type. Those participants with jobs requiring performance of tasks in both types of work processes were classified within the smelter subcohort. All analyses were stratified by work process type, as the two work processes differ significantly in particulate exposure in terms of concentration and sources [17], but also in terms of placement practices as the company exercised a post-hire job placement program for smelters, designed to place higher-risk individuals in less physically demanding jobs [14].

### Exposure Assessment

Average annual concentrations (mg/m^3^) for TPM were generated for distinct exposure groups within each work process to create a job exposure matrix. The estimates were based on more than 8000 personal industrial hygiene samples collected over 25 years by the company. Average annual PM_2.5_ concentrations were also estimated for the same distinct exposure groups, based on co-located measurements of PM_2.5_ and TPM personal exposures performed at 8 of the 11 facilities during 2010 and 2011. The exposure assessment and creation of the JEM are described in greater detail elsewhere [17]. Briefly, the JEM was based on personal samples collected randomly (rather than as part of a specific diagnostic evaluation or as targeted worst case). % of TPM composed of PM_2.5_ was estimated from the co-located TPM and PM_2.5_ measurements in the same subset of facilities. This estimate of % of TPM that is PM_2.5_ was used to derive model estimates of PM_2.5_ for distinct exposure groups in which no PM_2.5_ measurements were taken. A total of 80 distinct exposure groups were standardized for smelters and 101 for fabrication respectively. A further 10 groups with mixed smelter and fabrication exposures were also standardized. Workers in these 10 groups were not included in previous analyses of this cohort [14–16], but were included in the smelter subcohort in the current study leading to larger final sample size. The JEM was time invariant and no significant trends in exposure over time where observed for most jobs [18]. Personal exposures were assigned based on the job held at the beginning of each year. Time-varying exposures on the participant level were still possible as a result of changes in job held over time.

Confidence levels were assigned to both TPM and PM_2.5_ exposure values, with high level confidence assigned to those TPM exposure estimates derived from direct measurements as a data source as opposed to surrogate estimates or default values. High confidence PM_2.5_ estimates were derived from direct measurements of TPM and % PM_2.5_, or direct TPM measurements and estimated % PM_2.5_ based on information from similar exposure groups. The primary analysis was restricted to person-time with high confidence exposure estimates. Exposure was dichotomized into binary variables. In the absence of an appropriate external referent value as occupational limits are higher than more than 95% of the observed exposures (or not available for the case of PM_2.5_), we used exposure distribution based cut-offs as was the case in previous studies in this cohort [15, 16]. Binary exposure variables were created using the 10^*th*^ percentile of the respective TPM and PM_2.5_ average annual exposure distributions as a cut-off, while cut-offs at the 25^*th*^ percentile were assessed in a sensitivity analysis. We also examined a multi-category exposure variable, with values below the 10^*th*^ percentile as the referent category and four additional categories, divided based on the quartiles of the remainder of the exposure distribution.

### Outcome Assessment and Covariates

Incident IHD cases were ascertained from medical claims data through December 31st 2012. IHD cases were defined as subjects with insurance claims for relevant procedures (i.e., revascularization, angioplasty, bypass), hospitalization for 2 or more days, or face-to-face visits with International Classification of Diseases, Ninth Revision, admission codes for IHD (codes 410–414) or death from IHD (identified by International Classification of Diseases, Ninth Revision, codes 410–414 or International Classification of Diseases, Tenth Revision, codes I20–I25) while actively employed and without any previous incident IHD event. Termination of active employment with the company, transition to salary (as opposed to hourly worker) prior to the end of follow-up, and death from competing causes were possible censoring events.

Information was also available for age, sex, race, and job grade through employment records. Data on smoking status, height, and weight were collected at occupational health clinics located at each of the facilities, and availability varied by facility. Additionally, a time varying comprehensive health risk-score variable was available during follow-up. This claims-based variable was derived using a proprietary algorithm, initially to predict health expenditures, but has been found to be a strong predictor of a variety of health outcomes such as asthma, diabetes, hypertension and IHD in this cohort [19]. It is used in our analysis as a time-varying indicator of overall health status.

### Statistical Analyses

We previously reported presence of time varying confounding by health status affected by previous exposure in the smelter subcohort [15]. We used marginal structural Cox models and inverse probability weights to address this bias and the same approach is used in this study. Analyses for each of TPM and PM_2.5_ as the primary exposure were identical but for the exposure variables. Inverse probability exposure weights were estimated though a pooled logistic model for annual exposure. The exposure model included variables for attained age, sex, race, plant, job grade, smoking status, body mass index (BMI), the risk score variable (recoded into deciles), and a variable for exposure in the previous year. We used the model-predicted probabilities of exposure to assign subject specific time dependent weights to each person-year. The weights were equal to the cumulative product of the inverse of the model-predicted probability that each person received their observed exposure history through each time point. Estimation of inverse probability weights in described in greater detail elsewhere [20, 21]. Stabilized weights were estimated, with the time varying risk score variable excluded from the models for the numerator of the weights [22]. The same approach was used with multilevel logistic models in the case of the multi-category exposure variables.

Cox regression models were subsequently used with the weighted population, with attained age as the time-scale of interest. The same covariates were added as with the exposure models, with the exception of risk score, which was controlled for through weighting. Unweighted Cox models were used for multilevel exposure variables in the fabrication subcohort, as weighted models were not stable. Previous findings indicate that use of inverse probability weights to control for time varying health status does not affect estimates in the fabrication subcohort [15]. The proportional hazard assumption was tested using predictor and time interactions and Schoenfeld residuals. We also stratified Cox models by categorical variables for age. Further sensitivity analyses included evaluation of exposure cut-offs at the 25^*th*^ percentile, and restriction to person time with high confidence exposure values for the PM_2.5_ exposure metrics, which is more restrictive than the TPM metric; high confidence PM_2.5_ exposures accounted for 8,079 fewer person-years and 91 fewer cases than high confidence TPM exposures in the smelter subcohort and 6,667 fewer person-years and 56 fewer cases in fabrication.

Multiple imputation using the MI procedure in SAS, version 9.4, software (SAS Institute, Inc., Cary, North Carolina) was used to impute missing data for smoking (33% missing) and BMI (20% missing). BMI values were imputed using the expectation-maximization algorithm. Smoking status was subsequently imputed using the logistic regression method. All analyses were performed using SAS software (version 9.4, SAS Institute, Inc., Cary, North Carolina).

## Results

Table 1 summarizes demographic characteristics in the population, stratified by work process. Of 15,692 eligible workers at the participating facilities, 1,681 (10.7%) were excluded because of lack of high confidence exposure data. The final sample size for the smelter subcohort was 6,579 and 7,432 for the fabrication subcohort, with 502 (7.6%) and 556 (7.5%) IHD cases in each respective subcohort. The cohort was predominantly composed of white men. Baseline health characteristics were comparable in the two types of work processes.

**Table 1:** Demographic characteristics of a cohort of actively employed U.S aluminum workers, at start of follow up, stratified by work process.

Characteristic	Smelters (n = 6,579)				Fabrication (n = 7,432)			
	No %	Mean (SD)	Median	Range	No %	Mean (SD)	Median	Range
Year of hire			1985	1944–2010			1990	1949–2010
Male	6,058 (95.3)				5,623 (79.9)			
White	5,517 (86.8)				5,787 (82.2)			
Age		44.4 (10.4)				44.2 (10.2)		
BMI*^a^*		30.0 (5.1)				30.0 (5.6)		
Current Smokers*^a^*,	752 (24.7)				1,175 (29.7)			
Past Smokers*^a^*	1,035 (34.1)				956 (24.2)			
Risk Score			0.76	0.15–28.17			0.80	(0.15–27.67)

Abbreviations: BMI = body mass index; SD = standard deviation

^*a*^BMI data were only available for about 80% of the study population, while smoking status was available for about 66%.

The data presented here are based only on the available data.

**Fig 1:**
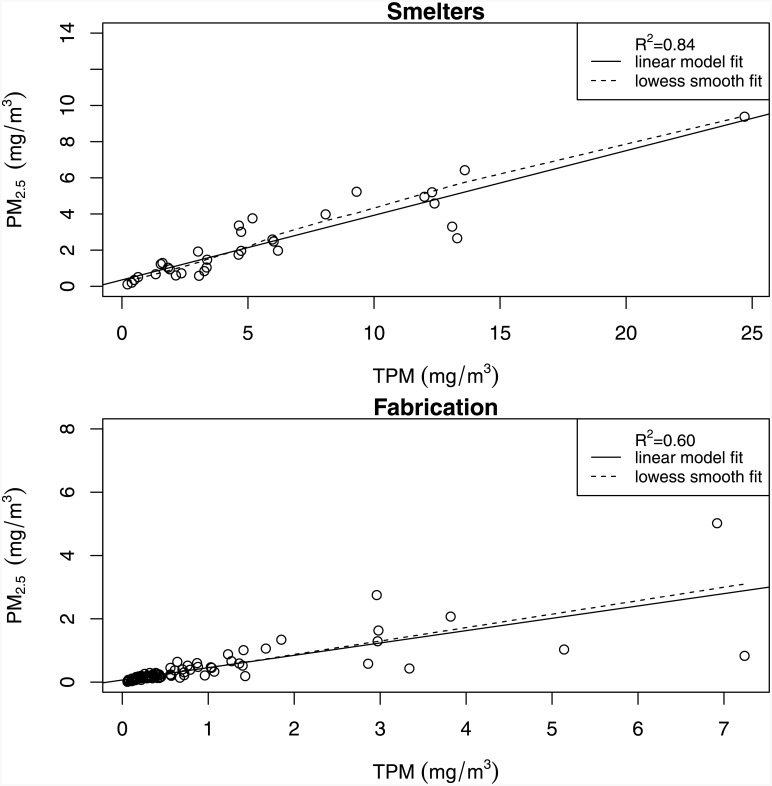
Scatterplot of TPM and PM_2.5_ for distinct exposure groups based on an aluminum industry JEM, for two different facility types: smelters (33 DEGs) and fabrication (99 DEGs). Solid lines represent a line of best fit, and the dashed line is a lowess line. Information in this figure is restricted to exposure groups with high confidence exposure values for both TPM and PM_2.5_.

**Fig 2:**
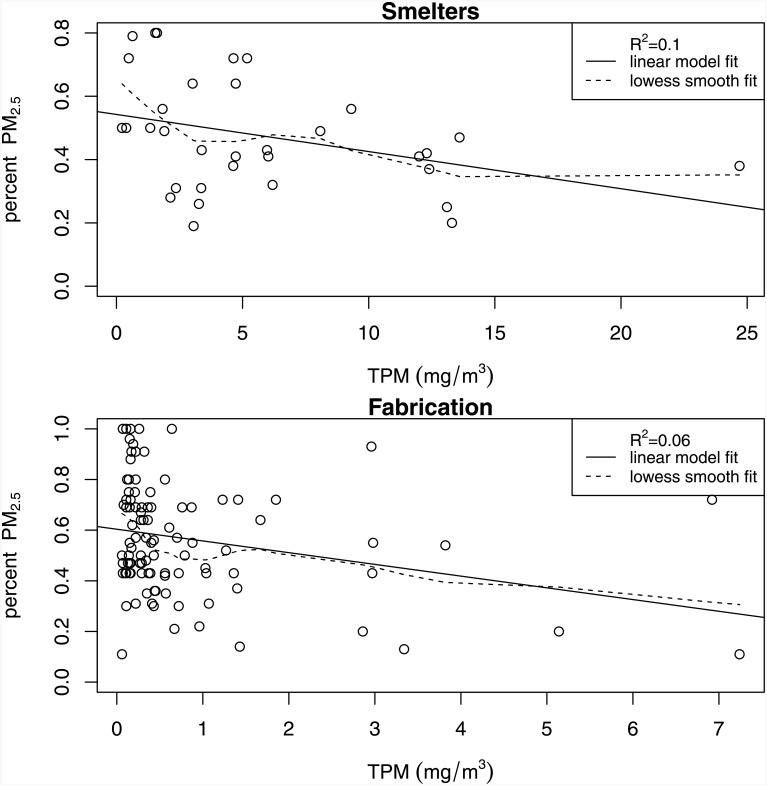
Scatterplot of TPM and percent PM_2.5_ for distinct exposure groups based on an aluminum industry JEM, for two different facility types: smelters (33 DEGs) and fabrication (99 DEGs). Solid lines represent a line of best fit, and the dashed line is a lowess line. Information in this figure is restricted to exposure groups with high confidence exposure values for both TPM and PM_2.5_.

**Fig 3:**
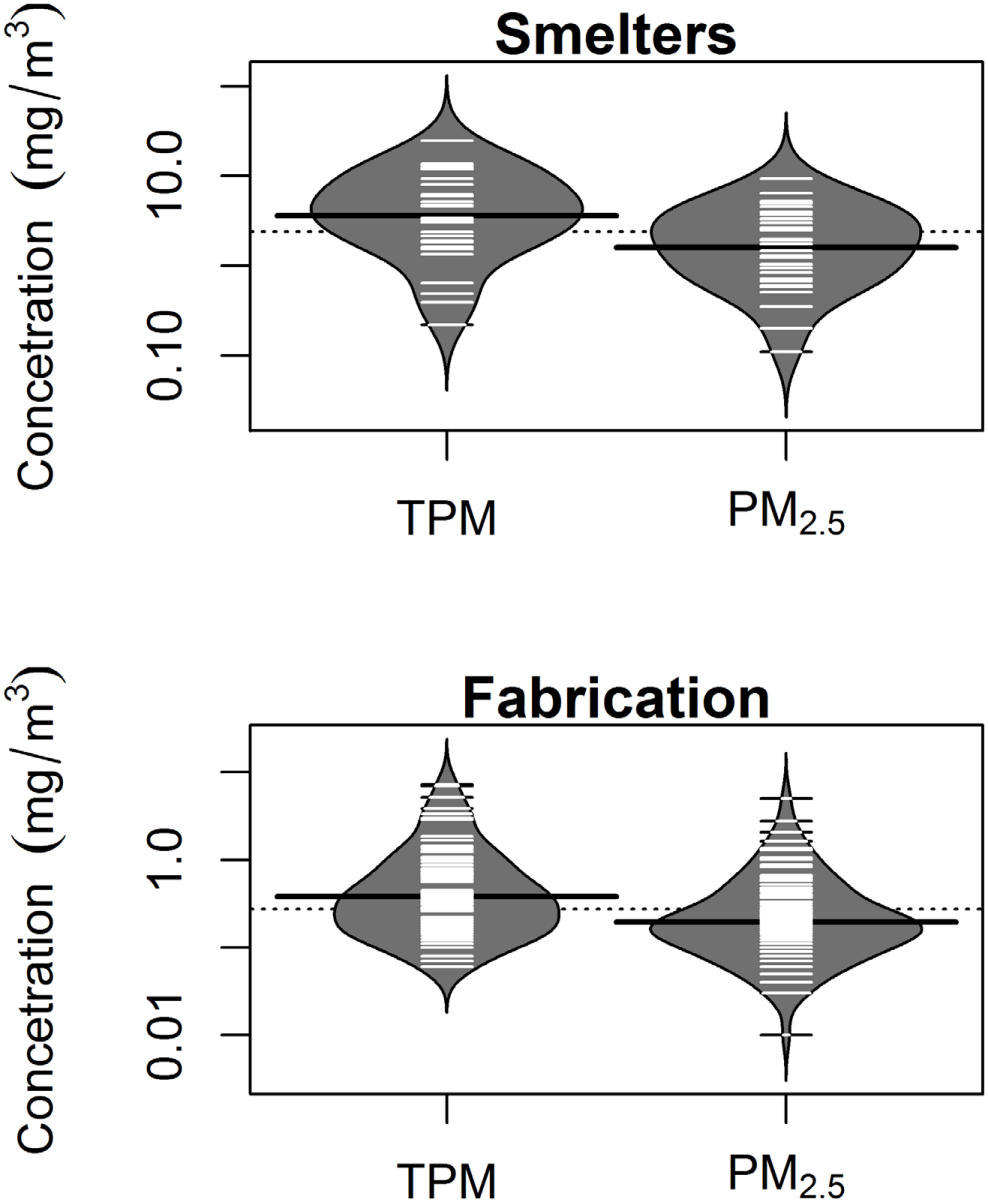
Beanplots of the distributions of TPM and PM_2.5_ concentrations for distinct exposure groups based on an aluminum industry JEM, for two different facility types: smelters and fabrication. The dark horizontal line represents the mean value for each distribution, while each white beanline represents a point for each distinct exposure group.

Average TPM and PM_2.5_ concentrations were greater in smelters compared to fabrication. Mean (SD) annual TPM exposures were 4.52 (3.70) mg/m^3^ and 0.57 (0.77) mg/m^3^ in smelters and fabrication respectively. Mean (SD) annual PM_2.5_ exposures were 1.98 (1.62) mg/m^3^ and 0.34 (0.50) mg/m^3^ in smelters and fabrication respectively. The R-squared between TPM and PM_2.5_ from the respective values for each distinct exposure group in the JEM was 0.84 in the smelter groups and 0.60 in the fabrication groups respectively (Fig 1). The R-squared on the person-time level (TPM and PM_2.5_ values per person-year in the cohort) was 0.87 in the smelters and 0.71 in fabrication. % PM_2.5_ was highly variable with respect to TPM concentrations with a slight indication for a downward trend in both facility types (Fig 2). R-squared values for TPM and % PM_2.5_ were much lower. Beanplots (a more detailed data distribution tool, alternative to the boxplot [23]) of the distributions of the two particle size concentrations in each work process are illustrated in (Fig 3). Pearson correlation coefficients for the binary exposure variables used in the primary analysis were 0.81 for the smelter subcohort and 0.74 for fabrication.

When comparing the two binary exposure variables, of the 4,326 person-years in the smelters that were below the 10^*th*^ percentile of PM_2.5_, 1,355 (31%) were above the 10^*th*^ percentile of TPM. In fabrication, of the 5,320 person-years that were below the 10^*th*^ percentile of PM_2.5_, 1,541 (29%) were above the 10^*th*^ percentile of TPM, while a further 722 person-years were below the 10^*th*^ percentile for TPM but above the 10^*th*^ percentile PM_2.5_.

The Hazard Ratio (HR) comparing always exposed above the 10^*th*^ percentile of TPM exposure in the smelters was 1.27 (95% confidence interval (CI): 0.90–1.80), while for PM_2.5_ the HR was 1.70 (95% CI: 1.11–2.60). The corresponding numbers in the fabricator subcohort were 1.25 (95% CI: 0.88–1.77) and 1.48 (95% CI: 1.02–2.13) respectively (Table 2).

**Table 2:** Hazard Ratios (95% CI) for the risk of IHD comparing exposure above and below the 10^*th*^ and 25^*th*^ percentile of exposure distributions for TPM and PM_2.5_ stratified by work process type.

Exposure	HR (95% CI)	
	Smelters	Fabrication
10^*th*^ percentile cut-off*		
TPM	1.27 (0.90–1.80)	1.25 (0.88–1.77)
PM_2.5_	1.70 (1.11–2.60)	1.48 (1.02–2.13)
25^*th*^ percentile cut-off^†^		
TPM	1.10 (0.85–1.43)	1.13 (0.85–1.50)
PM_2.5_	1.55 (0.90–2.67)	1.15 (0.89–1.48)

Abbreviations: TPM = total particulate matter; PM_2.5_ = particulate matter with an aerodynamic diameter <2.5 *μ*m; HR = hazard ratio; CI: confidence interval.

*10^*th*^ percentile cut-off values for TPM were 0.37 mg/m^3^ for smelters and 0.12 mg/m^3^ for fabrication, while 10^*th*^ percentile cut-off values for PM_2.5_ were 0.26 mg/m^3^ for smelters and 0.06 mg/m^3^ for fabrication.

^†^25^*th*^ percentile cut-off values for TPM were 1.54 mg/m^3^ for smelters and 0.16 mg/m^3^ for fabrication, while 25^*th*^ percentile cut-off values for PM_2.5_ were 0.72 mg/m^3^ for smelters and 0.12 mg/m^3^ for fabrication.

Higher exposure cut-offs (25^*th*^ percentile) resulted in attenuated HRs for both exposures but the results for PM_2.5_ were still elevated compared to TPM in the smelters. HRs from the multicategory exposure variable analysis for both exposures are summarized in (Table 3). Effect estimates were consistently higher for PM_2.5_ compared to TPM, in both subcohorts, but no monotonic exposure-response was evident. Effect estimates were similar for all quartiles of PM_2.5_ exposures compared to the referent category, while effect estimates for TPM exposures were lower for the two highest quartiles. Restriction to person time based on PM_2.5_ exposure confidence levels did not greatly affect results for TPM exposures. There was no evidence for violation of the proportional hazards assumption: global Chi-square tests for predictor×time interactions were negative and Schoenfeld residual plots did not indicate substantial deviations from proportionality.

**Table 3:** Hazard Ratios (95% CI) for the risk of IHD associated with categorical exposures to TPM and PM_2.5_ stratified by work process type.

Smelters*		Fabrication*	
Exposure category (mg/m^3^)	HR (95% CI)	Exposure category (mg/m^3^)	HR (95% CI)
TPM			
<0.372 (ref)	1.00	<0.120 (ref)	1.00
0.372–2.959	1.14 (0.74–1.75)	0.120–0.210	1.24 (0.88–1.73)
2.960–4.229	1.38 (0.95–2.02)	0.211–0.360	1.33 (0.92–1.93)
4.230–6.189	1.12 (0.73–1.72)	0.361–0.638	1.19 (0.79–1.79)
≥6.19	1.09 (0.72–1.64)	≥0.639	1.29 (0.90–1.84)
PM_2.5_			
<0.260 (ref)	1.00	<0.06 (ref)	1.00
0.260–1.284	1.62 (1.04–2.52)	0.06–0.1394	1.50 (1.05–2.16)
1.285–1.789	1.50 (0.87–2.60)	0.1395–0.2193	1.29 (0.86–1.92)
1.790–2.591	1.60 (0.98–2.64)	0.2193–0.3743	1.54 (1.03–2.33)
≥2.592	1.49 (0.89–2.49)	≥0.3744	1.51 (1.03–2.21)

Abbreviations: TPM = total particulate matter; PM_2.5_ = particulate matter with an aerodynamic diameter <2.5 *μ*m; HR = hazard ratio; CI: confidence interval.

*Results for smelters are from Cox models with inverse probability weights for the exposure while for fabrication results are from unweighted conditional Cox models, adjusting for time-varying risk score.

## Discussion

The objective of most industrial exposure monitoring programs is compliance with recommended guidelines or regulated exposure limits. OSHA has permissible exposure limit (PEL) for PM classified as particulates not otherwise regulated, or inert dust, for TPM and for the respirable fraction, with lung disease as the relevant health effect [13]. Mounting evidence, however, suggests that it is the smaller sized particles in the ambient environment that pose the greatest health risks, especially traffic-related PM_2.5_ and increased risk of cardiovascular disease [1, 3, 24, 25]. Effect estimates for IHD risk associated with occupational PM_2.5_ exposures in the aluminum industry were statistically significant and greater in magnitude than TPM exposures, indicating that PM_2.5_ is a stronger predictor of IHD, despite high correlation between the two exposures. The findings of the current study support the conclusion that fine particles are more strongly associated with cardiovascular disease in this occupational setting. The excess observed risk associated with particulate exposures is also of concern as there is no evidence of decreasing exposures over time in the aluminum industry [18, 26], and the observed exposures in this study are orders of magnitude lower than existing PELs.

We observed increased risk of IHD in relation to increased particulate exposure for both size fractions, but with higher and statistically significant effect estimates only for PM_2.5_ in both fabrication and smelters. Hazard Ratios for the risk of IHD associated with TPM above binary exposure cut-offs, set at a level much lower than the current OSHA PEL, were elevated, but 95% CIs included the null. When we looked at quartiles of exposure, the effect estimates did not increase monotonically. Absence of a clear monotonic exposure-response with either particle size fraction may be due to increased exposure misclassification at higher levels of exposure–workers in highly exposed jobs may be more likely to use a respirator, which was not accounted for in the JEM estimates for this study. Alternatively, a plateau in the magnitude of the HRs observed at higher exposures may be true [27], as has been observed in cigarette smoking and heart disease [28]. In addition to lack of information on respirator use, another potential limitation is the absence of PM_2.5_ measurements for a number of the exposure groups in the JEM. We restricted the analysis to person-time with exposure values based at least on TPM measurements (high confidence) to limit bias due to exposure misclassification. We have no reason to believe that exposure groups without direct PM_2.5_ measurements are differentially related to health outcomes. A further limitation of this study is the limited generalizability of quantitative effects of specific exposure levels as the exposure groups compared are specific to the exposure distribution in this cohort. However, the qualitative difference in the respective effect estimates for PM_2.5_ and TPM is nevertheless an important finding.

Most participants in this cohort did not develop the outcome or reach administrative end of follow-up in 2012, since subjects were censored at termination of active employment. Sensitivity analysis using censoring weights to account for potentially informative censoring did not greatly affect estimates in a previous study [15], so no adjustments were made in the current study. One of the strengths of this study was the wealth of information on many potential confounders not usually available in occupational studies. In particular, a comprehensive risk score variable [19] is a considerable strength. The risk score was created to predict health expenditures by the insurance carrier, using a proprietary algorithm. This time-varying measure of underlying health status allowed us to both examine and control for the presence of healthy worker survivor effect, an important bias in occupational epidemiology.

A high overall correlation between the two size fractions of PM might lead us to expect a small difference in the effect estimates for heart disease. The difference between HRs for TPM and PM_2.5_, however, was greater in the smelters where the correlation was higher than in fabrication. This difference between the differences in HRs might be because all the discordant pairs in the smelters involved person time below the PM_2.5_ exposure cut-off but above the TPM cut-off, whereas in fabrication the opposite was also true.

In the multilevel categorical analysis in the smelters, HRs comparing the risk of the two highest quartiles of exposure for TPM with a reference group were attenuated compared to the HRs for the two lowest quartiles. By contrast, the effect estimates for PM_2.5_ appeared more constant across the four exposure quartiles. This may suggest that TPM becomes a poorer surrogate for finer particles with increasing concentrations in the smelter subcohort.

Though the correlations between TPM and PM_2.5_ concentrations for specific exposure groups within each of the two facility types were high, PM_2.5_ as a % of TPM was quite variable across distinct exposure groups. The shapes of the distributions for the two particle size fractions were also similar within work process type. However, there appeared to be more exposure groups with high TPM values than with high PM_2.5_ values, especially in the smelters. This was consistent with an observed downward trend in the relationship between TPM and % PM_2.5_ concentration; % PM_2.5_ tended to be lower in exposure groups with higher TPM.

Smaller sized particles are thought to be more harmful to cardiovascular health, because they penetrate deeply into the lung, have increased surface area per mass and potentially increased reactivity. Furthermore, smaller particles are typically the product of combustion of fossil fuels as well as industrial processes that generate trace metals and organic compounds thought to be more relevant in promoting cardiovascular disease than nuisance dust [1, 3, 24]. In the occupational setting of the current study, particulate exposures arise from a variety of sources including inorganic dusts, metals, fumes, metalworking fluids, and lubrication oils. For example, grinding is more likely to produce larger particles, in contrast to welding or direct combustion processes in aluminum smelting that produce smaller particles [17]. A previous study of particle size within these same facilities, showed high variability in particle size distribution across different production areas, especially in the smelters [29]. The high variability in particle size distributions across jobs limits the utility of particles in one size fraction (e.g. TPM) as a surrogate for another size fraction (e.g. PM_2.5_).

The increasing health concerns about smaller sized particles extend to ultrafine particles (UFPs), <0.1 *μ*m in diameter [1, 25, 30–32]. Smelting processes in the aluminum industry produce high counts of particles in the ultrafine range [33, 34] but exposure assessment in this cohort suggests that UFPs may be generated in some areas of fabrication as well [29]. Future assessment of exposure to fine and ultrafine particles and their potential health effects, as well as a more in depth analysis of associations between specific chemicals and health outcomes in this industry will contribute towards the design of more effective exposure monitoring and control approaches.

### Conclusion

In this study, we observed an increased risk of incident IHD in relation to occupational exposure to PM in a prospective cohort study in the aluminum industry and, consistent with biologic plausibility, a higher risk for PM_2.5_ than for TPM. Based on these results, and given the highly variable distribution of particle sizes across jobs and processes in this industry, cardiovascular disease risk management would likely benefit from more targeted exposure monitoring.

## References

[1] Brook RD, Rajagopalan S, Pope CA, Brook JR, Bhatnagar A, Diez-Roux AV, et al Particulate matter air pollution and cardiovascular disease an update to the scientific statement from the American Heart Association. Circulation. 2010;121(21):2331–2378. 10.1161/CIR.0b013e3181dbece1 20458016

[2] Dockery DW, Pope CA, Xu X, Spengler JD, Ware JH, Fay ME, et al An association between air pollution and mortality in six US cities. New England Journal of Medicine. 1993;329(24):1753–1759. 10.1056/NEJM199312093292401 8179653

[3] Simkhovich BZ, Kleinman MT, Kloner RA. Air pollution and cardiovascular injury: epidemiology, toxicology, and mechanisms. Journal of the American College of Cardiology. 2008;52(9):719–726. 10.1016/j.jacc.2008.05.029 18718418

[4] Magari SR, Hauser R, Schwartz J, Williams PL, Smith TJ, Christiani DC. Association of heart rate variability with occupational and environmental exposure to particulate air pollution. Circulation. 2001;104(9):986–991. 10.1161/hc3401.095038 11524390

[5] Torén K, Bergdahl IA, Nilsson T, Järvholm B. Occupational exposure to particulate air pollution and mortality due to ischaemic heart disease and cerebrovascular disease. Occupational and Environmental Medicine. 2007;64(8):515–519. 10.1136/oem.2006.029488 17303673 PMC2078490

[6] Fang SC, Cassidy A, Christiani DC. A systematic review of occupational exposure to particulate matter and cardiovascular disease. International Journal of Environmental Research and Public Health. 2010;7(4):1773–1806. 10.3390/ijerph7041773 20617059 PMC2872342

[7] Pope CA III, Dockery DW. Health effects of fine particulate air pollution: lines that connect. Journal of the Air & Waste Management Association. 2006;56(6):709–742. 10.1080/10473289.2006.10464485 16805397

[8] Peng RD, Chang HH, Bell ML, McDermott A, Zeger SL, Samet JM, et al Coarse particulate matter air pollution and hospital admissions for cardiovascular and respiratory diseases among Medicare patients. JAMA. 2008;299(18):2172–2179. 10.1001/jama.299.18.2172 18477784 PMC3169813

[9] USEPA. U.S Environmetal protection Agency. National Ambient Air Quality Standards (NAAQS); 2012. Available from: http://www3.epa.gov/ttn/naaqs/criteria.html.

[10] Benke G, Abramson M, Sim M. Exposures in the alumina and primary aluminium industry: an historical review. Annals of Occupational Hygiene. 1998;42(3):173–189. 10.1093/annhyg/42.3.173 9684558

[11] Rønneberg A, Andersen A. Mortality and cancer morbidity in workers from an aluminium smelter with prebaked carbon anodes–Part II: Cancer morbidity. Occupational and Environmental Medicine. 1995;52(4):250–254. 10.1136/oem.52.4.250 7795740 PMC1128203

[12] Nieboer E, Thomassen Y, Chashchin V, Odland JO. Occupational exposure assessment of metals. Journal of Environmental Monitoring. 2005;7(5):411–415. 10.1039/b504183j 15934191

[13] OSHA. Occupational Safety & Health Administration. Chemical Sampling Information.; 2012. Available from: https://www.osha.gov/dts/chemicalsampling/data/CH_259640.html.

[14] Costello S, Brown DM, Noth EM, Cantley L, Slade MD, Tessier-Sherman B, et al Incident ischemic heart disease and recent occupational exposure to particulate matter in an aluminum cohort. Journal of Exposure Science and Environmental Epidemiology. 2014;24(1):82–88. 10.1038/jes.2013.47 23982120 PMC4045503

[15] Neophytou AM, Costello S, Brown DM, Picciotto S, Noth EM, Hammond SK, et al Marginal Structural Models in Occupational Epidemiology: Application in a Study of Ischemic Heart Disease Incidence and PM2. 5 in the US Aluminum Industry. American Journal of Epidemiology. 2014;180(6):608–615. 10.1093/aje/kwu175 25125691 PMC4157703

[16] Brown DM, Petersen M, Costello S, Noth EM, Hammond K, Cullen M, et al Occupational Exposure to PM2. 5 and Incidence of Ischemic Heart Disease. Epidemiology. 2015;26(6):806–814. 26079662 10.1097/EDE.0000000000000329 PMC4741411

[17] Noth EM, Dixon-Ernst C, Liu S, Cantley L, Tessier-Sherman B, Eisen EA, et al Development of a job-exposure matrix for exposure to total and fine particulate matter in the aluminum industry. Journal of Exposure Science and Environmental Epidemiology. 2014;24(1):89–99. 10.1038/jes.2013.53 24022670 PMC4067135

[18] Noth E, Liu S, Cullen M, Eisen E, Hammond SK. 0122 Approaches to developing exposure estimates that reflect temporal trends in total particulate matter in aluminium smelters. Occupational and Environmental Medicine. 2014;71(Suppl 1):A14–A14.

[19] Hamad R, Modrek S, Kubo J, Goldstein BA, Cullen MR. Using “Big Data” to Capture Overall Health Status: Properties and Predictive Value of a Claims-Based Health Risk Score. Plos One. 2015;10:e0126054 10.1371/journal.pone.0126054 25951622 PMC4423900

[20] Hernán MÁ, Brumback B, Robins JM. Marginal structural models to estimate the causal effect of zidovudine on the survival of HIV-positive men. Epidemiology. 2000;11(5):561–570. 10955409 10.1097/00001648-200009000-00012

[21] Cole SR, Hernán MA. Constructing inverse probability weights for marginal structural models. American Journal of Epidemiology. 2008;168(6):656–664. 10.1093/aje/kwn164 18682488 PMC2732954

[22] Robins JM, Hernan MA, Brumback B. Marginal structural models and causal inference in epidemiology. Epidemiology. 2000;11(5):550–560. 10955408 10.1097/00001648-200009000-00011

[23] Kampstra P. Beanplot: A boxplot alternative for visual comparison of distributions. Journal of Statistical Software. 2008;28(1):1–9.27774042

[24] Hopke PK. New directions: reactive particles as a source of human health effects. Atmospheric Environment. 2008;42(13):3192–3194. 10.1016/j.atmosenv.2008.03.002

[25] Franck U, Odeh S, Wiedensohler A, Wehner B, Herbarth O. The effect of particle size on cardiovascular disorders—The smaller the worse. Science of the Total Environment. 2011;409(20):4217–4221. 10.1016/j.scitotenv.2011.05.049 21835436

[26] Donoghue AM, Frisch N, Ison M, Walpole G, Capil R, Curl C, et al Occupational asthma in the aluminum smelters of Australia and New Zealand: 1991–2006. American Journal of Industrial Medicine. 2011;54(3):224–231. 10.1002/ajim.20925 21298697

[27] Stayner L, Steenland K, Dosemeci M, Hertz-Picciotto I. Attenuation of exposure-response curves in occupational cohort studies at high exposure levels. Scandinavian Journal of Work, Environment & Health. 2003;29:317–324. 10.5271/sjweh.737 12934726

[28] Pope CA III, Burnett RT, Krewski D, Jerrett M, Shi Y, Calle EE, et al Cardiovascular mortality and exposure to airborne fine particulate matter and cigarette smoke shape of the exposure-response relationship. Circulation. 2009;120(11):941–948. 10.1161/CIRCULATIONAHA.109.857888 19720932

[29] Liu S, Noth EM, Dixon-Ernst C, Eisen EA, Cullen MR, Hammond SK. Particle Size Distribution in Aluminum Manufacturing Facilities. Environment and Pollution. 2014;3(4):79–88. 10.5539/ep.v3n4p79 26478760 PMC4607067

[30] Delfino RJ, Sioutas C, Malik S. Potential role of ultrafine particles in associations between airborne particle mass and cardiovascular health. Environmental Health Perspectives. 2005;113:934–946. 10.1289/ehp.7938 16079061 PMC1280331

[31] Weichenthal S. Selected physiological effects of ultrafine particles in acute cardiovascular morbidity. Environmental Research. 2012;115:26–36. 10.1016/j.envres.2012.03.001 22465230

[32] Ostro B, Hu J, Goldberg D, Reynolds P, Hertz A, Bernstein L, et al Associations of Mortality with Long-Term Exposures to Fine and Ultrafine Particles, Species and Sources: Results from the California Teachers Study Cohort. Environmental Health Perspectives. 2015;123:549–556. 10.1289/ehp.1408565 25633926 PMC4455590

[33] Thomassen Y, Koch W, Dunkhorst W, Ellingsen DG, Skaugset NP, Jordbekken L, et al Ultrafine particles at workplaces of a primary aluminium smelter. Journal of Environmental Monitoring. 2006;8(1):127–133. 10.1039/B514939H 16395469

[34] Debia M, Weichenthal S, Tardif R, Dufresne A. Ultrafine particle (UFP) exposures in an aluminium smelter: Soderberg vs. prebake potrooms. Environment and Pollution. 2012;1(1):2–11.

